# An Overview of Functionalized Graphene Nanomaterials for Advanced Applications

**DOI:** 10.3390/nano11071717

**Published:** 2021-06-29

**Authors:** Andrea Maio, Ivana Pibiri, Marco Morreale, Francesco Paolo La Mantia, Roberto Scaffaro

**Affiliations:** 1 Research Unit INSTM—Department of Engineering, University of Palermo, Viale delle Scienze, 90128 Palermo, Italy; 2 Department of Biological, Chemical and Pharmaceutical Sciences and Technologies (STEBICEF), University of Palermo, Viale delle Scienze, 90128 Palermo, Italy; ivana.pibiri@unipa.it; 3 Faculty of Engineering and Architecture, Kore University of Enna, Cittadella Universitaria, 94100 Enna, Italy; marco.morreale@unikore.it

**Keywords:** graphene, graphene oxide, graphene quantum dots, energy, fuel cells, sensors, tissue engineering, catalysis, drug release, water treatment

## Abstract

Interest in the development of graphene-based materials for advanced applications is growing, because of the unique features of such nanomaterials and, above all, of their outstanding versatility, which enables several functionalization pathways that lead to materials with extremely tunable properties and architectures. This review is focused on the careful examination of relationships between synthetic approaches currently used to derivatize graphene, main properties achieved, and target applications proposed. Use of functionalized graphene nanomaterials in six engineering areas (materials with enhanced mechanical and thermal performance, energy, sensors, biomedical, water treatment, and catalysis) was critically reviewed, pointing out the latest advances and potential challenges associated with the application of such materials, with a major focus on the effect that the physicochemical features imparted by functionalization routes exert on the achievement of ultimate properties capable of satisfying or even improving the current demand in each field. Finally, current limitations in terms of basic scientific knowledge and nanotechnology were highlighted, along with the potential future directions towards the full exploitation of such fascinating nanomaterials.

## 1. Introduction

Graphene is a one-atom-thick carbon layer with a honeycomb-like structure, which was isolated in 2003 for the first time. It can be considered as the basic unit of the so-called graphene nanomaterials [1], a category that encompasses—among others—graphene nanoplatelets (GNP), constituted by few graphene sheets stacked to each other, and graphene oxide (GO), i.e., its oxidized form. Graphene and GNP display a hexagonal honeycomb lattice made of sp^2^ hybridized carbons, which can be regarded as a 2D planar, conjugated structure endowed with strong aromaticity. GNP and their multilayered, micrometric counterpart (graphite) can be easily converted into either graphene (via exfoliation) or GO (via oxidation). The structure of this latter can be regarded as a double honeycomb, bearing the tunable presence of both sp^2^ and sp^3^ hybridized carbon atoms, thus resulting in the coexistence of aromatic and oxygenated domains. These latter, causing the distortion of 2D lattice, endow GO with a wrinkled, wavy, and crumpled texture [2,3,4,5,6]. Recent studies on GO structural modelling unveiled its metastability, thus suggesting that GO should not be regarded as a static material with a given set of functional moieties [2,3,7]. Despite their differences, all the revisions to former models agree on considering GO as a family of closely related materials, or a composite material itself, of which the composition, dependent on several conditions used within synthesis and purification steps, evolves over time [2,3,7].

More in general, physicochemical features of graphene-based nanomaterials are strongly affected by the type of fabrication route (including synthetic protocols and subsequent treatments) and raw sources [8].

This study will show how the target applications of graphene nanomaterials varies as a function of their oxidation level, which unambiguously determines physicochemical properties of such nanomaterials.

In this regard, the biocompatibility of graphene-based materials was found to increase upon augmenting the hydrophilicity, i.e., the O/C ratio [9]. Highly oxygenated GO samples, especially those having small lateral size, demonstrated good cytocompatibility and remarkable capability of stimulating cell adhesion, differentiation, and signaling, relying on highly hydrophilic character and wrinkled texture [2,3,4,5,6]. By contrast, electrical and thermal conductivity increase upon decreasing O/C ratio and lattice defects, thus making sp^2^-conjugated graphene nanomaterials more suitable for these latter applications.

**Figure 1:**
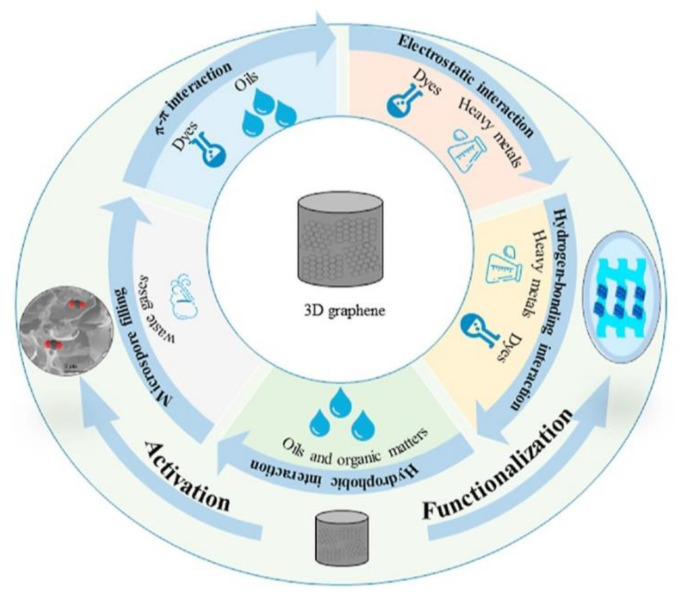
Pictorial representation of the mechanisms involved during removal of various pollutants by using three-dimensional graphene materials. Reprinted with permission from [10], Copyright Elsevier, 2020.

**Figure 2:**
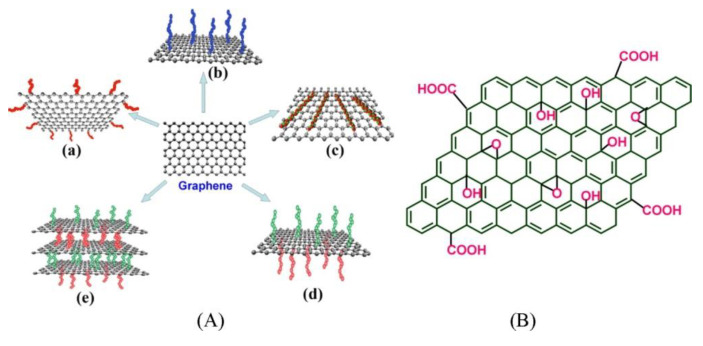
(**A**) Functionalization possibilities for graphene: (**a**) edge functionalization, (**b**) basal plane functionalization, (**c**) noncovalent adsorption on the basal plane, (**d**) asymmetrical functionalization of the basal plane, and (**e**) self-assembling of functionalized graphene sheets. (**B**) Chemical structure of graphene oxide. Reprinted with permission from [11]. Copyright 2013, American Chemical Society.

Therefore, GO attracts enormous interest especially for applications in biomedicine, and surfactants or lubricants, while graphene and GNP are often more suitable than GO for applications in flexible electronics and energy storage/conversion. All the graphenic materials demonstrate exceptional ability to trap other substances, thus being lately proposed as promising materials for environmental applications. In this latter case, the possibility to prepare graphene with tunable physicochemical characteristics is a key factor. In fact, modulation of oxidation level, size, and surface chemistry via derivatization allows for the removal of different classes of air and aqueous pollutants, including either polar or nonpolar aliphatic and aromatic molecules, waste gases, refractory organics, heavy metals, salts, oils, and so on. A generic representation of the mechanisms that graphene materials exploit for the removal of various pollutants is provided in Figure 1 [10]. GO is more prone to further derivatization reactions that encompass both non-covalent and covalent functionalization approaches, while graphene, due to the prevalence of sp^2^-conjugated carbons, is preferably functionalized via π–π stacking complexation or introducing specific moieties onto edge or basal planes, as depicted in Figure 2 [11].

All the graphenic materials can be doped in an easy, tunable way, thus being suitable for energy storage applications [12,13].

Graphene nanomaterials can be conveniently integrated into organic (e.g., polymers) and inorganic matrices and, thus, processed into thin films, sheets, fibers, membranes, foams, aerogels, and so on, leading to the possibility to attain 0D, 1D, 2D, and 3D materials, which can be even endowed with graded or hierarchical architecture [12]. In the last decade, hybrid structures of graphene and other nanocarbons, such as carbon nanotubes (CNTs), were proposed, aiming to exploit synergistic effects in mechanical, thermal, and electrical performance [14,15,16].

## 2. Materials Engineering

Over the last years, numerous applications in materials engineering have been conceived involving graphene or its derivatives, due to the interesting features in terms of mechanical, thermal, and electrical properties [17,18,19,20,21,22,23].

In the following, some relevant examples of actual materials engineering applications are described.

### 2.1. High Thermomechanical Performance Materials

One of the most desired goals in materials engineering is to achieve lightweight systems with enhanced mechanical and thermal resistance properties. From this point of view, the rise in graphene has sparked groundbreaking research, which has paved the way for quite promising developments [24]. Some relevant examples are cited in the following.

Gan et al. [25] succeeded in functionalizing graphene with D-glucose via esterification and preparing a nanocomposite with poly(vinyl alcohol) (PVA) and poly(methyl methacrylate) (PMMA) as matrices. They found that the functionalized graphene dispersed homogeneously in the matrices and led to significant improvements of thermomechanical properties, which are thought to be due to the strong hydrogen bond interactions between the polymer blend and D-glucose moieties attached onto fillers.

Chhetri et al. [26] functionalized GO nanoparticles with 3-amino-1,2,4–triazole (TZ) in KOH as catalyst and integrated such nanohybrids into epoxy resin by solution mixing. The resulting nanocomposites exhibited improved mechanical and thermal resistance. In detail, tensile strength and elastic modulus enhanced about 30% in comparison to those of composites containing pure GO, and fracture toughness doubled; TGA tests showed an approx. 30 °C increase in the onset degradation temperature.

Yong et al. [27] fabricated nanocomposites from polyimide (PI) 3-aminopropylethoxysilane functionalized GO, via in situ polymerization and thermal imidization, finding significant improvements in the mechanical and thermal properties in comparison to the neat PI and depending on the functionalized GO amount: a bare 1.5 wt.% allowed them to obtain approx. 130% increment of the tensile modulus, 80% increase in the tensile resistance, and 200% increase in the thermal conductivity.

Bian et al. [28] prepared a complex system involving high-density polyethylene grafted with maleic anhydride (HDPE-g-MA), GO functionalized with ethyilenediamine (GO-EDA) and oxidized carbon nanotubes (MWCNTs-COOH). GO-EDA and MWCNTs-COOH were coupled by L-aspartic acid, and the resulting hybrid network was integrated into HDPE-g-MA via melt compounding. Tensile, impact, and DMA characterization indicated that the properties underwent significant improvements in comparison to the neat polymer matrix, while TGA showed that a bare 0.75 wt.% of hybrid loading allowed for an increase in the maximum decomposition temperature of about 11 °C.

Another important field where the use of graphene and derivatives may be of great interest is the packaging industry. In this case, the main applications are food packaging, which requires biodegradable materials due to the increasing environmental concerns related to waste disposal, and electronic packaging, which requires good barrier properties against gases and, especially, water vapor. In the latter case, the use of graphene seems particularly promising, thanks to its capability to improve mechanical properties, chemical durability, and barrier properties [29,30]. An example of application was provided by Gui et al. [31,32], who covalently immobilized polyurethane-imide and 4-allyloxy-biphenyl-4-ol onto GNP, with functionalization performed in ethanol for 30 min under ultrasonication, through the setting up of covalent bonding and π–π interactions. Nanocomposites were then prepared with vinyl silicone resin prepolymer. They found that 1 wt.% of functionalized GNP allowed for the obtaining of a dramatic increase in the mechanical properties, i.e., about 500% of the original tensile strength, while 2 wt.% enhanced the elastic modulus up to 1000%. At the same time, higher functionalized GNP percentages (10–15%) lead to very remarkable improvements of the thermal conductivity (from 16 up to 38 times the original conductivity of the resin).

**Figure 3:**
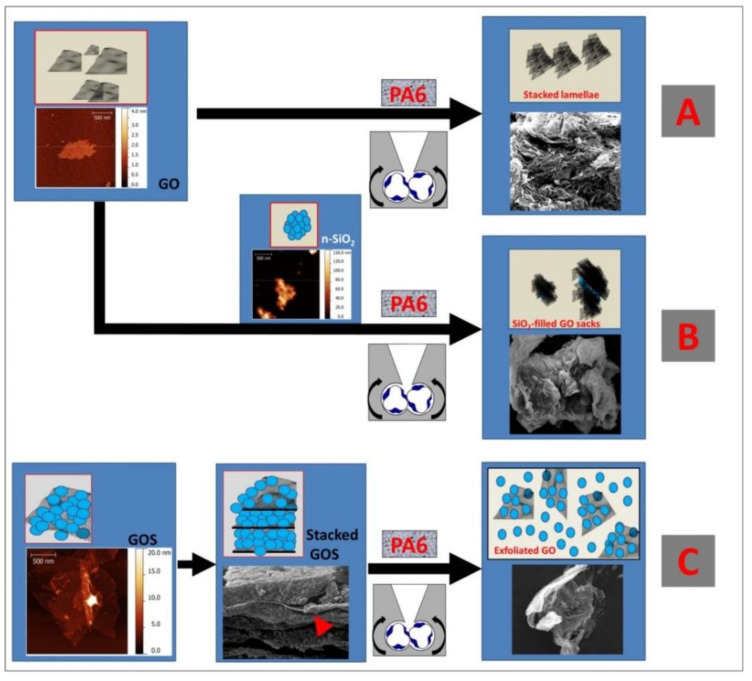
Schematics of the pathway followed and processing-structure relationship for nanocomposites A (direct melt mixing of PA6 and GO), nanocomposites B (melt mixing of PA6, GO, and nanosilica), nanocomposites C (synthesis of lasagna-like self-assembled GOS nanohybrids and melt mixing with PA6. AFM of nanomaterials and SEM of stacked GOS and polymer-based nanocomposites are provided together with the pictorial representation. Reprinted with permission from [36]. Copyright Elsevier, 2016.

Non-covalently functionalized reduced graphene oxide nanoplatelets were used to grow in situ SiO_2_ nanoparticles, thus giving rise to hierarchical structures. These latter were found to act as reinforcing agent for a hydrogenated nitrile butadiene rubber matrix, providing strong enhancement of both the static and the dynamic mechanical properties [33]. GO-SiO_2_ nanohybrids can also be prepared via covalent bonding [34,35]. Such nanoplatforms, prepared in water by either Fischer esterification or ethylendiamine-mediated coupling, were successfully tested as reinforcing fillers for thermoplastic polymers, including polyamide 6 (PA6) [36], polylactic acid (PLA) [37], and thermoplastic polyurethane (TPU) [38]. Figure 3 pictorially describes the outstanding exfoliation level of PA6 nanocomposites containing GO-silica nanohybrids (C), when compared to those containing GO (A) or a physical mixture of GO and silica (B) [36]. 

The intriguing thermal and heat transfer properties, which can be imparted by the presence of graphene and derivatives, also suggest the use for conventional working fluids, obviously in order to improve their thermal conductivity. For instance, Sadri et al. [39] developed a route to fabricate highly water-soluble, functionalized GNP, based on free radical grafting of gallic acid onto GNP at 80 °C, 12 h, reflux conditions. The obtained nanofluid, containing just 0.05 vol% of nanoparticles, was found to possess approx. 25% higher thermal conductivity in comparison to the reference fluid.

### 2.2. Antibiofouling Coatings

Biologically generated fouling (biofouling) on marine equipment is a well-known issue related to the undesired growth of algae, plants, and microorganisms on submerged surfaces [40]. Although it can also happen outside of marine environments, it is in those scenarios that it becomes more significant and annoying, blocking accesses and reducing the hydrodynamic performance of crafts (thus increasing fuel consumption) [41]. Therefore, it is important to coat marine-submerged surfaces with suitable anti-biofouling products. Those, especially over the last years, need to be not only effective, but also with toxicity and environmental impact features as low as possible. For this reason, currently, the most used antifouling agents are based on Cu_2_O and organic, environment-friendly biocides. On the other hand, biodegradable antifouling coatings would be of great interest, since they would lead, through degradation, to a “polishing” of the biofouling layer and to a “self-renewal” of the involved surfaces [42]. Unfortunately, the actual application of these biodegradable coatings is undermined by the low values of degradation rate as well as of the adhesion strength. For instance, biodegradable polyurethanes (PU) and polylactides (PL) can find application in this field due to their good mechanical properties and biocompatibility, but on the other hand, especially with regard to PUs, they typically show slow hydrolysis kinetics and poor adhesion characteristics [42]. Additionally, in this case, the literature suggests that the incorporation of graphene may significantly help in making the product more biodegradable and at the same time improve the antibacterial activity, while still keeping a reasonably long service life. Ou et al. [43] prepared a biodegradable polyurethan, containing units of poly(L-lactic acid) as soft segments, and graphene. Phenol-derivatized graphene was used as an initiator for ring-opening polymerization of L-lactic acid and the resulting polymer grafted with graphene underwent condensation polymerization with diphenylmethane-diisocyanate to form polyurethan. They found it to be a suitable coating for smooth surfaces, with better antifouling behavior and hydrolysis capability in comparison to the neat PU.

### 2.3. Lubricants

It is well known that most of the lubricants used for steel-based machinery and items are organic-based; on the other hand, environmental impacts considerations would require using more ecologically friendly systems, for instance based on water (which are already used in other fields). Unfortunately, water is not suitable as a lubricant for steel-based systems. Therefore, research has been carried out in order enhancing the tribological features of water, for instance by dispersing nanoparticle additives; in this context, graphene has been studied for use as a water-lubricating additive [40]. One of the main limitations is due to carbon nanomaterials being hydrophobic; therefore, surface treatments are needed in order to impart hydrophilicity to this class of additives; at the same time, significant friction and wear reduction must be guaranteed.

One possibility is represented by carbon nanotube-ionic liquid (IL) [44,45] and graphene-IL [45,46] hybrid lubricants, which showed intriguing friction and wear performance. Graphene-IL hybrid systems were added to polyethylene glycol (PEG) [47] to exploit the synergistic effect of graphene and ILs in reducing friction, with graphene covalently functionalized with methyl imidazolium oleate ([MIM][OL]), hexafluorophosphate ([MIM][PF_6_]), and bis(salycilato)borate ([MIM][BScB]) by means of suitable propyltrimethoxysilyl bridges. This led to significantly improved anti-wear behavior (increasing according to the above listing order) in comparison to the neat ILs-PEG systems. Significant anti-friction and wear properties were also found in systems prepared from GO and PEG subjected to γ-radiolysis in water [48].

Finally, also fluorinated graphene (FG) was taken into consideration, due to its excellent properties; on the other hand, its hydrophobicity strongly reduces its actual applicability. In this view, a trial was performed [49] to enhance its hydrophilicity via melt functionalization with urea. It was found that, after 4 h at 150 °C treatment, urea was covalently grafted onto FG, and that even small amounts of UFG (1 mg/mL) lead to significant reductions (up to approx. 60%) in the wear rate.

### 2.4. Flexible Electronics

**Figure 4:**
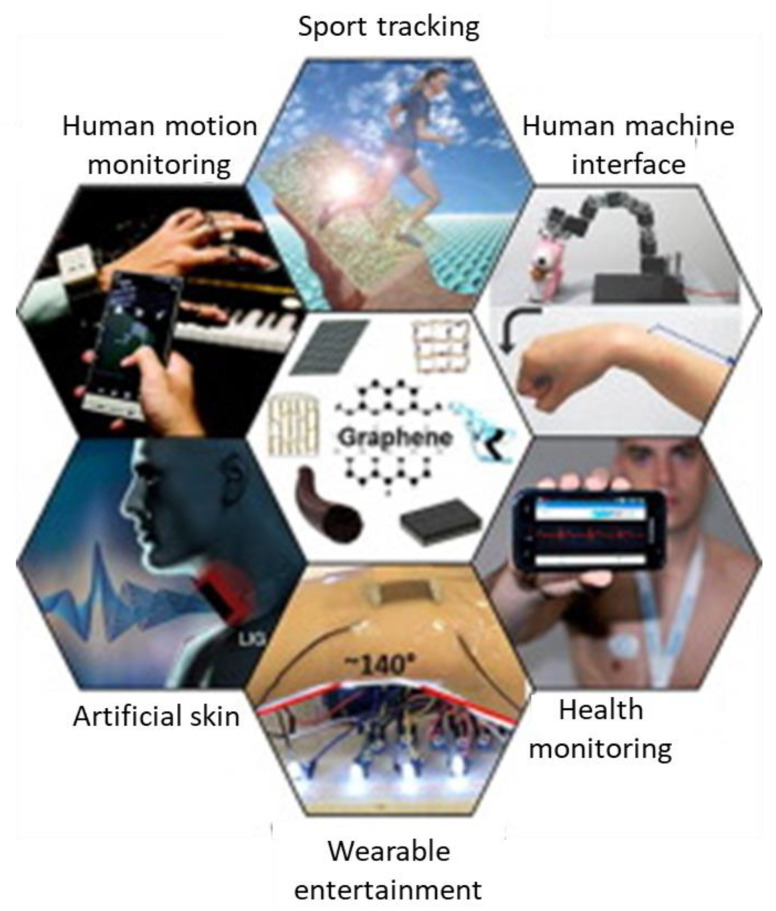
Examples of possible applications of graphene-based flexible electronics. Reprinted with permission from [52]. Copyright Elsevier, 2019.

Flexible electronics is the terminology usually adopted to indicate electronic devices mounted on flexible substrates to obtain flexible electronic circuits [50,51], which are, therefore, of any desired shape and, at the same time, can be bent during the operative use, finding application in consumer electronics and medical devices, as depicted in Figure 4 [52]. More specifically, such a device should have features such as bendability, stretching ability, twistability, and stable electronic performance, and a flexible substrate is fundamental in obtaining a light, thin, and ductile device. In this perspective, functionalized graphene and derivatives can find application as fillers in a polymer matrix, improving the dielectric constant and keeping good flexibility and overall mechanical properties. Furthermore, it may be interesting to investigate the potential application of graphene in self-healing flexible electronics: self-healing substrates capable of repairing or recovering themselves after being subjected to use-related damages.

The literature reports some examples. Manna and Srivastava [53] prepared, via solution mixing, nanocomposites based on carboxylated nitrile rubber (XNBR) and hexadecylamine-functionalized graphene nanosheets (GNS-HDA). They found some very promising results, such as an approx. 60% increase in elongation at break and tensile strength, 13% reduction in the elastic modulus (and thus increased flexibility), 14-fold increased dielectric constant, and enhanced thermal stability, thus providing a suitable material for flexible dielectric devices.

Wu et al. [54] prepared, through Diels-Alder chemistry, a composite based on polyurethane (PU), covalently bonded to RGO, showing good tenacity and IR-laser self-healing properties. They found that a bare 0.5 wt.% led to an improved stress at break equal to 36 MPa and, in particular, the ability to recover after breaking most of the initial properties, through laser irradiation (980 nm) for 60 s.

Another study also involving Diels-Alder chemistry was performed by Li et al. [55], who prepared polyurethane composites covalently cross-linked with functionalized RGO. First, they modified GO with furfurylamine in water (60 °C, 12 h) obtaining FRGO sheets, and then, they covalently crosslinked it with linear PU bearing furfuryl moieties by Diels-Alder cycloaddition reactions. They obtained, in summary, composites with improved mechanical properties and thermal stability and, moreover, healing efficiency by 5-minute microwave irradiation.

**Figure 5:**
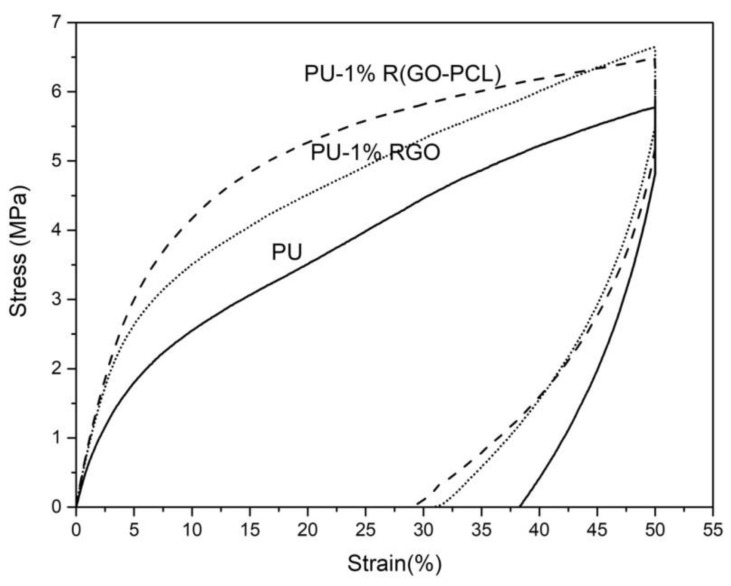
Thermomechanical cyclic behavior of neat PU and the related nanocomposites. Reprinted with permission from [56]. Copyright Elsevier, 2019.

Lotfi Mayan Sofla et al. functionalized GO with polycaprolactone (PCL) and then added it to polyurethane to prepare shape memory nanocomposites [56]. Chemical bonding of PCL to GO was accomplished by the esterification reaction between OH groups of PCL and COOH groups of GO nanosheets. Results demonstrated that functionalized GO increased the shape recovery and mechanical properties of nanocomposites, as showed in Figure 5.

### 2.5. Optical Limiters

The use of lasers is now widespread in several different fields, including medical treatments, optical communications, chemical measurements, and materials processing. On the other hand, the use of lasers also requires the utilization of suitable protections for sensitive optical apparatuses and human eyes, which involve the development of highly efficient optical limiters (OLs) [40,57].

OL behavior has been found in some systems, such as organic dyes, metal nanoparticles, quantum dots, and carbon-based ones [58]. In particular, graphene has been attracting considerable interest due to its OL properties deriving from its sp^2^-conjugated carbon lattice and the linear dispersion of electronic band structure, which results in a nonlinear optical (NLO) characteristic [40,57]. Furthermore, such behavior was also found in graphene-related composites [58].

For instance, Xu et al. [59] covalently functionalized RGO with three conjugated polymers containing carbazole, tetraphenylethylene, and phenyl moieties. They found an improved dispersion stability of RGO and, somehow unexpectedly, OL behavior strongly dependent on the type of polymer grafted onto nanoplatforms. In particular, two of them showed excellent OL performance, since they even responded to a 4 μJ input laser intensity.

Recently, decorating GO with nanoparticles such as metals and metal oxides is an emerging trend for the development of OL materials. In this context, Ren et al. [57] adopted hydrothermal method to prepare TiO_2_/rGO nanohybrids that showed enhanced NLO and OL features with respect to starting components. Moreover, such a performance was found to improve upon decreasing the size of TiO_2_. With a similar approach, Nancy et al. [60] developed a novel, green synthetic approach to decorate GO lamellae with laser-induced silver nanoparticles. The resulting nanohybrids gathered NLO behavior with antimicrobial properties, thus potentially serving as antibacterial OLs.

GO was incorporated into a silico-phosphate glassy matrix via the sol-gel method to construct films capable of exerting optical limiting functions for femtosecond laser pulses in the infrared-B spectral region [61]. Additionally, fluorographene can be conveniently used in OL applications, and in this regard, Stathis et al. [62] proposed a series of *N*-octylamine-modified fluorographenes with tunable NLO response.

## 3. Energy Engineering

### 3.1. Electronics and Optoelectronics

The presence of highly mobile electrons in the graphene lattice obviously suggests intriguing properties for applications in the field of electronics [14].

For instance, systems from GO and 1-pyrene carboxylic acid formed by π–π complexation revealed good potential for being employed in photovoltaics, photochemistry, and photocatalysis [63].

GO-spyropyranpyrene nanocomposites were also studied [64], showing high tendency to Zn^2+^ coordination and good potentiality for photovoltaics and optics.

Furthermore, π–π stacking can be used for fabrication of OLEDs from conjugated polymers and GO [65,66,67,68,69,70,71]. For instance, functionalized poly(methylmethacrilate) (PMMA) derivatives in combination with GO and RGO were studied. The nanocomposites (prepared in DMF or NMP) showed enhanced mechanical, optical, and thermal properties, for possible application in OLEDs and photovoltaics. Moreover, poly (diallyldimethylammonium chloride) (PDDA)/GO hybrid nanocomposites processed in water were also prepared, showing promising properties for fuel cell applications.

Functionalization of graphene derivatives is particularly important for optoelectronics applications.

Yao et al. [72] adopted Suzuki coupling reaction to covalently functionalize graphene with thiophene and polythiophene (PTH), thus introducing improved electron delocalization and reduced band gap energy (in comparison to neat tiophene and PTH).

Ji et al. [73,74] studied the functionalization of graphene with different organic ligands and different complex functional groups, in order to control the work function (WF) of graphene sheets obtained via CVD, obtaining products with enhanced conductivity and good dispersibility in several solvents. They also proved the applicability of such functionalized graphene nanosheets as interlayers in organic field effect transistors, in order to control the WF of metallic electrodes, with an overall device performance enhancement.

### 3.2. Electrochemical Supercapacitors

The increasing market request for energy storage materials to be used in automotive sector and microelectronics is posing research to a new challenge, in order to improve the performance of supercapacitors. These devices can be used either for energy storage or high-power supply, storing energy, since they gather strong electrostatic attraction and fast charge transfer [40]. Their capabilities strongly rely on the extended active surface area, which is related to wettability and eventual porosity of the electrode materials; therefore, some features of rGO, such as high specific area and limited hydrophilicity, enable its use for the manufacturing of suitable supercapacitor devices. Furthermore, such a performance can even be improved by non-covalent coupling of rGO with suitable, electroactive compounds that enhance its dispersibility in water. Literature reports several examples [75,76,77,78], including Polyindole and Ag nanoparticles grafted on GO and RGO or several water soluble organic dyes, including methyl green (MG), sulfanilic acid azocromotrop (SAC), 9-anthracene carboxylic acid (ACA), and bisphenol A (BPA), non-covalently bonded onto GO and RGO. In all cases, significant enhancements of specific capacitance, rate capability, and cycling stability were found, ranging from 72% capacitance retention (up to 10–20 A/g) and 12% capacitance decay after 5000 cycles in the case of MG (similar values in the case of SAC), to 81% retention and 10% decay after 4000 cycles, in the case of BPA [40].

In order to increase porosity (as previously hinted), suitable 3D structures have been engineered in form of aerogels, using GO and anthraquinone, or GO and poly(diallyldimethyl ammonium chloride) [40]. Enhanced electrochemical energy storage and improved absorption of lithium ions were found, respectively, suggesting a worthwhile use as ultra-fast capacitors and long-life anode electrodes (respectively) [65,79]. The use of GO can be worthwhile also in conjunction with conducing polymers such as poly(o-methoxyaniline) (POMA): the covalently grafted POMA/GO nanocomposites demonstrated electrochemical capacitance as high as 422 F g^−1^ at 0.5 A g^−1^ current density and satisfactory durability (less than 5% capacitance loss after 1000 cycles) [80].

Recently [81], a 2D porous polymer was prepared by self-assembly of triazine-based compounds, to obtain N-enriched conducting polymer nanosheets. The outcomes has pointed out some interesting features, such as the possibility to modulate the thickness by varying the rGO amount, a high specific surface area (up to almost 2000 m^2^/g) and superior performance in comparison to other conducting polymer-based electrode materials.

In most cases, controlled functionalization of rGO surfaces is necessary, in order to avoid restacking and to increase wettability. In this direction, ionic liquids (ILs) seem to be particularly suitable, thanks to their good electrochemical and thermal stability and ionic conductivity. They have been used [82,83] in combination with graphene and CNTs in the development of supercapacitor devices.

In general, one of the main problems related to the use of graphene sheets (which, as briefly discussed, are suitable to prepare dielectric polymer composites, thanks to layered surface and high specific area) is due to dispersion and re-aggregation (which, in turn, leads to loss of dielectric properties). Surface functionalization, as already suggested, is the most straightforward way to improve exfoliation and dispersion of the graphene sheets in polymers, as well as their compatibility with the polymer matrix, avoiding direct contact between the nanosheets and, thus, keeping good dielectric properties.

**Figure 6:**
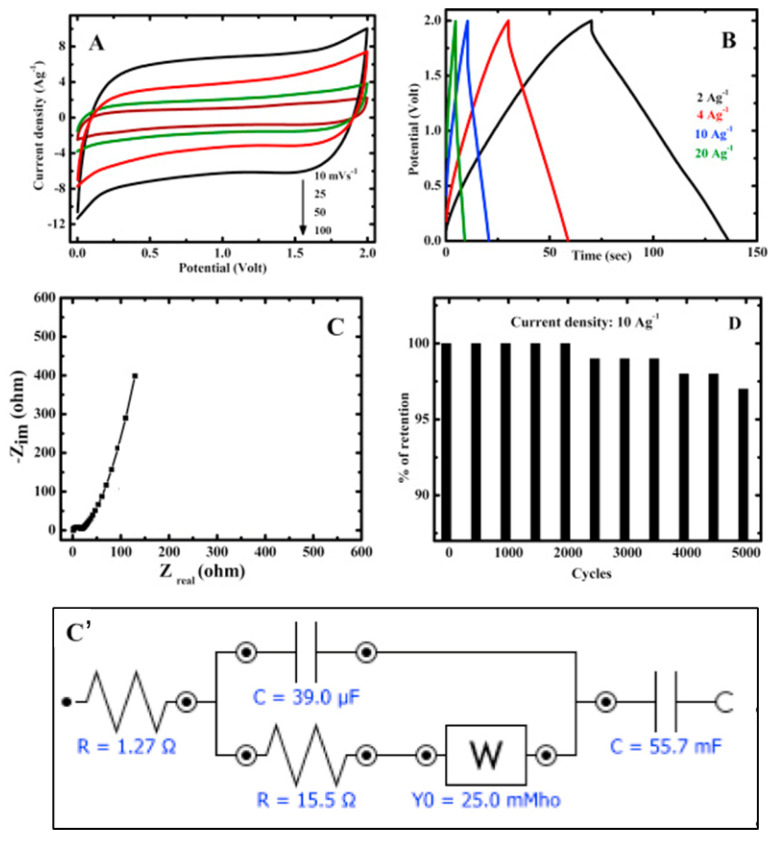
Cyclic voltammogram (**A**), charge-discharge profile (**B**), and Nyquist plot (**C**) of rGO-Im-IL-based aqueous symmetric supercapacitor. Plot illustrating the cycling stability of the supercapacitor is shown in (**D**). (**C’**) is the equivalent circuit used to fit the impedance data of (**C**). Reprinted with permission from [85], Copyright Elsevier, 2016.

For instance, diglycidyl ether of bisphenol-A (DGEBA) has been successfully grafted onto GO to obtain DGEBA-functionalized rGO, which showed an almost 10 times higher dielectric constant in comparison to neat epoxy, thanks to efficient dispersion in epoxy matrix [84]. Bag et al. [85] covalently prepared functionalized GO sheets by using hydrophilic imine-terminated ionic liquid (Im-IL), increasing the wettability, the interlayer distance, and, as a consequence, the potential window of the derived electrode as well as the specific capacitance. The obtained supercapacitor showed excellent properties, such as an energy density of almost 37 Wh/kg (at a power density of 2 kW/kg) and a cycling stability of just 3% specific capacitance loss after 5000 charge–discharge cycles, as provided in Figure 6.

Another recent study by Youan et al. [86] explained a novel strategy to obtain nanoarchitectures based on porous graphene frameworks (PGFs), through in situ derivatization of rGO, reacting with 4-iodobenzene diazonium salt in water, and subsequent Yamamoto coupling. The results showed significantly improved properties, such as specific capacitance and cycling stability, attributed to stable microporous structure with very high ion-accessible surface area.

### 3.3. Fuel Cells

It is well known that the international research is focusing on alternative energy sources in replacement for traditional, fossil-fuel-based ones, and fuel cells are definitely among the most interesting. At the same time, many technological problems and shortcomings have strongly hindered their development so far. For instance, the use of direct methanol fuel cells (DMFCs), having Pt composites as anode catalysts [87], has some drawbacks in the cost and amount of Pt needed. Another major problem is the usually slow kinetics of the oxygen reduction reaction (ORR).

Therefore, the possible application of graphene and derivatives in this field has been investigated as well.

For instance, as a possible way to reduce the use of Pt, Hosseini et al. [88] prepared reduced GO nanosheets functionalized with amine groups of magnesium phyllosilicate clay (RGC). Functionalization was performed in water, and then, RGC was added with Pt nanoparticles, finding an improved catalytic activity in comparison to Pt nanoparticles in the methanol oxidation reaction.

In order to improve the ORR kinetics, some studies on graphene-based catalysts, or metallic catalysts supported by functionalized graphene, have been carried out [89,90,91,92].

**Figure 7:**
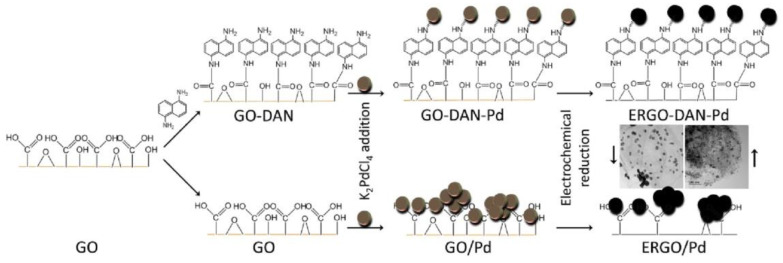
Preparation of ERGO/Pd and ERGO-DAN-Pd. Reprinted with permission from [93] Copyright Elsevier, 2016.

Park et al. [93] prepared GO functionalized with 1,5-diaminonaphtalene (DAN), which can stabilize metal nanoparticles; thus, they used the functionalized graphene as support for Pd nanoparticles (see Figure 7). The obtained material showed enhanced electrocatalytic ORR activity and long-term stability in comparison to standard Pt-based catalysts.

### 3.4. Solar Cells

The world demand for more efficient solar energy devices and, in particular, for improved solar cells is a topical subject and gives rise to great interest. Graphene and derivatives have been taken into account due to their properties. Studies have been performed on both traditional polymeric solar cells (PSC) and some new-generation ones such as, for instance, dye-sensitized solar cells (DSSC).

With regard to the former category, there is evidence of a detailed study from Vinoth et al. [94] on the use of hybrid materials made of graphene, polyaniline (PANI), and a ruthenium (Ru) complex. They compared the properties of a traditional device based on the PANI-Ru metallopolymer assembly with those of an alternative device where the metallopolymer assembly was covalently grafted on rGO sheets. The results were quite encouraging, since the open circuit potential (V_oc_) was measured to be six times higher, while the short circuit current density (J_sc_) was two times higher.

With regard to novel DSSC systems, there is a significant number of results that may be cited, with the common goal to achieve performance enhancements of the components and the overall device [95,96]. For instance, one of the main issues is related to the encapsulation, in particular to prevent the possible leakage of organic, volatile liquid electrolytes. To mitigate the problem, solvent-based organic volatile liquid electrolytes have been replaced by alternatives such as ionic liquids, solid-state charge transport materials, and polymer gels [97,98]. Ionic liquids are particularly interesting by virtue of their physicochemical stability coupled with high electrical conductivity, and they can be used in the form of gel. A study by Brennan et al. [99] showed that electrolytes containing a small percentage (1 wt.%) of graphene sheets in IL enhanced the efficiency of devices up to 25%. Another study [100] focused on the effects of using GO functionalized with two different ionic liquids (quaternary ammonium cations, differing in the final chain length), finding that an tributyl ammonium-based ionic liquid GO system experienced improved stability and, above all, an almost 10% improved conversion efficiency in simulated solar light tests.

**Figure 8:**
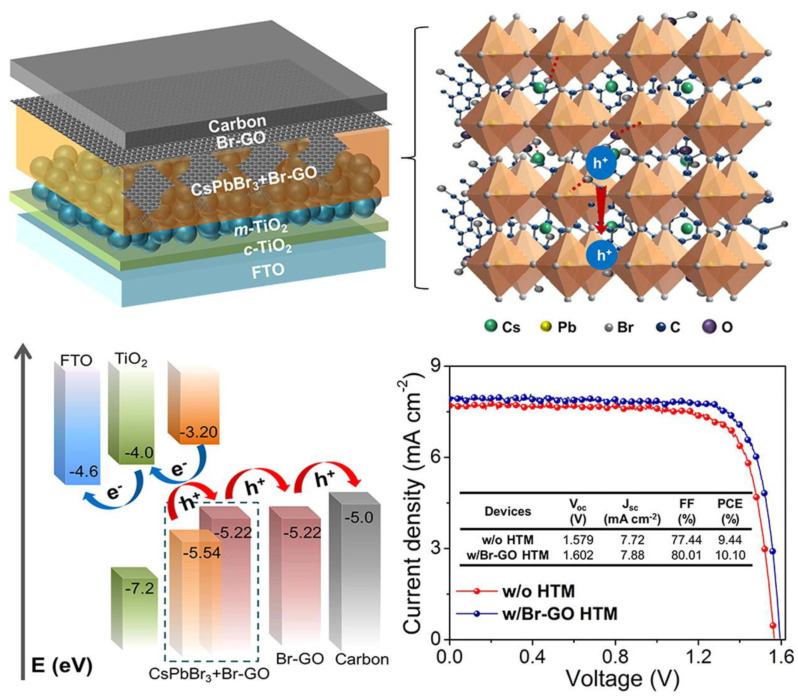
Structure and main properties of solar cells based on CsPbBr_3_ and Br-GO. Reprinted with permission from [101]. Copyright Elsevier, 2021.

Recently, Sun et al. [101] used brominated GO (Br-GO) to boost charge extraction in perovskite solar cells based on CsPbBr_3_ (Figure 8). The authors discovered that integrating Br-GO allowed for maximization of the grain size and hole mobility of perovskite film, while reducing defects and charge recombination events. Hence, the devices fabricated showed high efficiency and exceptional tolerance to variations of moisture and illumination.

Very recently, graphene quantum dots (GQD), obtained by either progressive cutting of graphene oxide or partial pyrolysis of organic matter, are emerging as promising candidates for dye-sensitized solar cells. Their escalation is ascribed to several reasons. In fact, GQD display fascinating features, such as quantum confinement, ecofriendly nature, large surface area, photoluminescence, and conversion of UV into visible light. They can be easily functionalized with oxygenated, nitrogenated, and halogenated groups, thus allowing tunable and tailored surface chemistry [102,103]. Moreover, many suitable dyes can be rapidly adsorbed onto GQD surface without compromising the sp^2^ network, thus modifying electron-carrying properties and conductivity of GQD [102,103]. Noteworthy is the possibility to enhance light absorption until covering the full solar spectrum from 300 to 900 nm. Dye-sensitized solar cells based on functionalized GQD ensure excellent performance in terms of electron transfer while minimizing back-electron transmission recombination at the photoelectrode/electrolyte edge [103].

Furthermore, electronic charges originating from molecular adsorption can be conveniently exploited in photovoltaic devices. In fact, GQDs/silicon heterojunction, with its exceptional power conversion efficiency (PCE) of 16.55%, still remains to date the most performing photovoltaic cell ever [103].

### 3.5. Solar Thermal Fuels

The search for alternative and renewable energy sources has relied on the immense potential coming from the sun from decades, but only a very small fraction of this potential has been successfully exploited. One of the most recent and novel pathways to deploy solar energy is represented by solar thermal fuels. These are, typically, metastable molecular forms able to release almost their entire stored energy (heat) through reconversion to stable forms, following an external stimulus. Although they are intriguing thanks to their capability to store and release energy, typical shortcomings and limitations are related to low storage capabilities and low stability (degradability) under prolonged irradiation. Research is, therefore, focusing on the improvement of the properties and to overcome most of these issues. In general, optimal solutions for these materials would rely on a high density of cromophore molecules, covalently attached to suitable surfaces. One example is based on azobenzenes cromophores (AZO) covalently bonded to carbon nanostructures, such as carbon nanotubes (CNTs), which showed good properties [104]. On the other hand, reduced graphene oxide (RGO) seems even more promising as nanostructured support, being able to anchor a plenty of photoactive compounds. An example is provided by the work from Luo et al. [105], where AZO molecules were covalently attached to graphene nanosheets, mainly relying on intermolecular H-bonds and obtaining a nano-template with a long-term storage lifetime (τ_1/2_ higher than 30 days) and remarkable storage capacity (more than 100 Wh/kg). The characterization also reported good performance for 50 cycles and the feasibility to at least 4.5 years of utilization, thus providing an encouraging basis for the development of improved solar thermal energy storage systems.

## 4. Sensors

One of the most important issues in clinical diagnostics is related to the feasibility of efficient methods to quantitatively detect biomarkers and therapeutic targets in biological tissues [40].

Some typical biomarkers are secreted extracellular proteins, which are often glycosylated and, therefore, called glycoproteins. These are relevant in several biological processes in living organisms [106]. However, the typical paths for glycoprotein recognition usually involve expensive and time-consuming procedures, with the ensuing hindered possibility of wide application [107,108,109,110,111].

On the other hand, molecular imprinting is an easy and cheap molecular detection technique that ensures high selectivity [40] and relies on molecularly imprinted polymers (MIPs), i.e., polymeric tailor-made affinity materials [112,113]. The integration of MIPs with electrochemical devices can provide a useful and interesting approach for the development of biochemical sensors endowed with outstanding selectivity and sensitivity [40]. However, imprinting of biomacromolecules having high molecular weight and complex structures, e.g., proteins and polypeptides, can be extremely difficult, even considering their scarce solubility in organic solvents. To address these shortcomings, graphene and especially GO have been recently evaluated as candidate supporting materials for the manufacturing of surface molecularly imprinted composites [114,115]. In fact, thanks to their high surface-to-volume ratio, they can accommodate a high number of recognition sites, with significant electrical conductivity, thus providing a versatile platform for constructing electrochemical sensors with outstanding sensitiveness toward large size biomacromolecules.

Huang et al. [116] proposed a way to covalently modify graphene for the production of biosensors. More in detail, they adopted a sol-gel strategy to functionalize GO with boronic acid (BGO), thus preparing a BGO molecularly imprinted polymer composite with ovalbumin (OVA) as the glycoprotein template. Then, via the pH control, it was possible to extract the OVA from the electrode surface, allowing it to detect the OVA of biological fluid samples dissolved in PBS, thanks to the different electrical response of the sensor as a function of OVA concentration, using [Fe(CN)_6_]^3−^/^4−^ as probe. The electrochemical tests showed high selectivity for the OVA in comparison to other similar glicoproteins (such as, for instance, bovine hemoglobin).

Other well-known strategies to selectively detect biomacromolecular markers are based on the use of the relative antibody (Ab), also known as immunoglobulin (Ig), capable of detecting specific antigens.

Eissa et al., for instance, reported on the covalent functionalization of graphene with electrochemically reduced carboxyphenyl diazonium salt [117]. Then, a further functionalization of the monolayer graphene was performed by immobilization of OVA Ab. They monitored the charge transfer resistance as a function of the OVA concentrations, finding high sensitivity of this functionalized CVD graphene substrate device.

Another important application of sensors using biomarkers would regard the detection of the prostate-specific antigen (PSA), usually exploited to early diagnose prostate cancer. In this view, Barman et al. chemically functionalized GO with glucose in water and, then, electro-deposited gold nanoparticles on the RGO surface, in order to improve the immobilization of the Ab anti PSA, detecting significant current change at 10 ng mL^−1^ of PSA [118].

Another class of biosensors for clinical diagnostics relates to impedimetric biosensors for DNA detection, which have several advantage points in comparison to other biosensors, such as simplicity and higher sensitivity [119,120]. Basically, their activity relies on the possibility to precisely monitor the eventual variations in interfacial electrical charge occurring during hybridization with target DNA.

Urbanová et al. [121] constructed the first type of graphene-based biosensor for impedimetric detection of DNA. More in detail, they prepared an efficient, low-cost thiofluorographene sample via sonochemical exfoliation of fluorinated graphite and subsequent reaction with sodium hydrosulfide in DMF.

However, DNA, polypeptides, and glycoproteins can be useful even for other analysis tasks. Some examples are hydrogen peroxide, widespread as an oxidizing agent, or glucose, important for diagnosing diabetes.

In this context, Ren et al. [122] derivatized GO via covalent immobilization of triethylenetetramine to prepare glucose biosensors with IO_4_^−^ oxidized glucose oxidase (GOx) through layer-by-layer self-assembly and subsequent deposition onto gold substrate. The attained sensor displayed outstanding performance in terms of sensitivity, stability, fast responsivity, and linearity.

Similarly, You et al. [123] decorated GO with Pd nanoparticles, using 5-amino-1,3,4-thiadiazole-2-thiol molecules as spacers and, then, applied such a nanohybrid for biochemical sensing of H_2_O_2_ and glucose oxidase. Cyclic voltammetry and chronoamperometry evidenced a linear responsivity up to 6.5 mM, with a detection limit of 10 μM for H_2_O_2_. When used as a glucose biosensor, instead, this device showed remarkable sensitivity even in the presence of physiological interferences [123].

Electrochemical functionalization is another, very effective controlled method to covalently functionalize graphene in order obtaining suitable biosensors.

For instance, a recent paper [124] reports on electrochemical functionalization through anodic oxidation to prepare amino-functionalized graphene dendrimers as enzymatic biosensors that demonstrated high suitability for enzymes, proteins, DNA, antibodies, antigens, and other biomolecules. The authors showed that the functionalized graphene device could immobilize the horseradish peroxidase (HRP) enzyme and, thus, provide highly sensitive detection of hydrogen peroxide, which is important in biological processes as well as in chemical, pharmaceutical, and food industries. Overall, the device showed not only enhanced sensitivity and electrocatalytic activity, but also stability.

One of the latest trends in clinical diagnostics regards the possibility to develop biosensors capable of detecting cancer biomarkers in the early stages, aiming at preventing cancer-related deaths significantly. In this context, very recently, Bai et al. [125] functionalized rGO with folic acid molecules, via π–π complexation, with the purpose to gather the excellent targeting features of folic acid with the electrocatalytic performance of graphene into a unique electrochemical sensing platform. The reaction between GO and folic acid was successfully performed in DMSO/water solution at pH 7 for 12 h at 37 °C. Such relatively inexpensive nanohybrid material demonstrated high sensitivity to a cancer biomarker, with current responses in a linear concentration range from 6 to 100 pM and a detection limit of 1.69 pM.

Another intriguing application for such biosensors regards dairy cattle health management. It has been found that a high level of circulating serum β-hydroxybutyrate (BHBA) in cows is symptomatic of diseases related to immune system, reduced milk production, poor fertility, etc. [126]. Electrochemical detection of BHBA can be performed by using enzyme β-hydroxybutyrate dehydrogenase (HBDH) associated with coenzyme nicotinamide adenine dinucleotide phosphate (NADP^+^) [127]. This approach would be faster and more convenient than the traditional spectroscopic techniques [128,129]. In this context, Veerapandian et al. [130] reported on the fabrication of a GO-based sensor, through covalent immobilization of Ru (II), NADP^+^, and HBDH onto nanosheets. Such a hybrid device showed remarkable ability to recognize BHBA, exhibiting, in particular, an improved redox behavior with a current sensitivity of 22 ± 2.51 μA mM^−1^ and demonstrated effectiveness and a quick (<1 min) response time.

Mao et al. [131] prepared polypyrrole/graphene oxide nanosheets functionalized with different hydrophilic polymers, aiming to increase their dispersibility in water. Furthermore, electrochemical performance of such hybrid sensors proved to be easily tunable by changing the type of hydrophilic polymer attached. When deposited onto electrodes, such nanohybrids enabled the quick detection of dopamine (DA) and ascorbic acid (AA). Among the polymers tested, using polyacrylates such as polyacrylamide and polyacrylic acid, allowed selectively distinguishing DA from AA in their mixture at certain concentrations, based on the different affinity towards electron donor amides of the former and electron acceptor carboxylic acids of the latter.

As previously mentioned, selective ion sensing is useful in applications such as food quality control, environmental monitoring, and clinical diagnostics. For instance, Olsen et al. [132] reported the preparation of functionalized graphene-18-crown-6 ether hybrid systems capable of selectively detecting even micro-molar amounts of K^+^ ions. Notably, their selectivity proved to be unaffected even in the presence of other cations (Ca^2+^, Li^+^, Na^+^, NH_4_^+^), since crown ether endowed the structure with pores that have the same size as potassium ions [132].

Another study [133] demonstrated that a low-temperature hydrothermal process can be effectively used to prepare porphyrin–graphene hybrids that serve as biocompatible and reusable sensors. Such nanomaterials were able to detect nitric oxide (NO), with good sensitivity (3.6191 μA μM^−1^) and electrocatalytic properties (0.61 V) in comparison to starting, reference materials.

**Figure 9:**
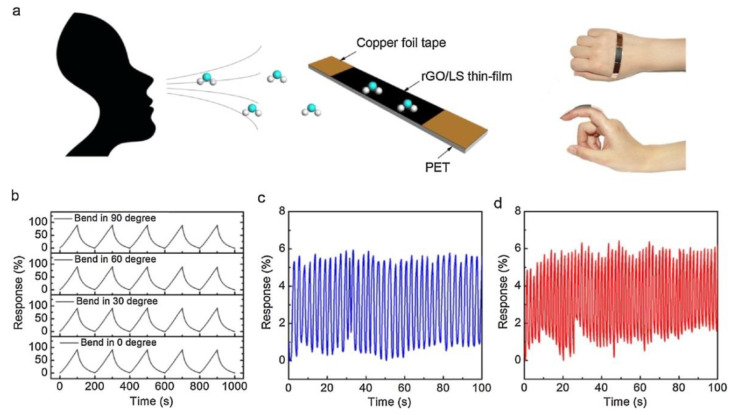
(**a**) Schematic image of a flexible respiratory frequency transducer and the photos of a flexible transducer attaching on human skin. (**b**) Bending test of a flexible respiratory frequency transducer in 0, 30, 60, and 90° for 5 cycles. (**c**,**d**) Recorded response-time curves of human breathing frequency before and after vigorous exercise. Reprinted with permission from [135]. Copyright Elsevier, 2017.

Recently, it was also shown that enzyme electrodes can be prepared by ion beam sputtering deposition [134,135]. More in details, graphene nanodots were encaged into porous gold and then non-covalently bonded with enzymes that were previously functionalized with pyrene molecules, aiming to exploit π–π interactions [134]. The obtained caged nanodots displayed with high sensitivity and reusability. GO and lignosulfonate (LS) were assembled by π–π stacking in water, in order to obtain systems that proved to be sensitive to relative humidity, suitable for manufacturing of respiratory frequency transducers, characterized by low cost, flexibility, and suitability to application in touchless user interfaces to detect human breath, as shown in Figure 9 [135].

## 5. Biomedical Engineering

The properties of GO, its biocompatibility, and the capability to be functionalized in a number of different, application-tailored ways and targets suggest it as a feasible option for biomedical engineering, in particular for drug delivery carriers, tissue engineering, and biosensors [136,137,138]. Some relevant studies in the field are reported in the following.

### 5.1. Drug Delivery

As previously stated, graphene-based nanomaterials have a good capability of carrying drugs, as well as a satisfactory biocompatibility.

Kavitha et al. [139] tested poly(4-vynil pyridine) (P4VP) functionalized GO for drug delivery and antimicrobial use; in particular, they tested the carrier properties of an anti- cancer drug, camptothecin (CPT), as well as its release by pH modulation, finding good in vitro anti-cancer properties; furthermore, interesting antimicrobial properties in testing against Escherichia coli and Staphylococcus areus were also found. These results merge with the relatively low cost and the good biocompatibility and stability of the obtained system.

Important applications may also deal with the delivery of anticancer drugs. For instance, doxorubicin (DOX) is a well-known anticancer drug, and its delivery from GO-based platforms has been investigated [140,141] due to the strong affinity between the drug and GO and the large specific area of this latter. Both studies relied on suitable functionalization of GO, including the use of surfactants such as hydroxyethyl cellulose and polyanionic cellulose, chitosan, and dextran. The results showed that higher DOX loading than usual could be achieved, as well as efficient delivery to the cancel cells. In addition to these applications in cancer treatment, biomaterials for photo/NIR chemotherapy can be obtained via the π–π complexation of DNA or other biomolecules with GO, which can be easily carried out in water [142,143]. Nanohybrids constituted by DOX and GO functionalized with Pluronic F127 molecules demonstrated good potential for the signal pathway of tumor-targeting therapy [144].

Finally, with regard to antibiotic delivery, a study has been reported concerning the possibility to form GO complexes with several Schiff bases in green solvents such as water and ethanol, obtaining biomedical devices with strong activity against Gram+ and Gram− bacteria [40]. Beyond this, because of their high affinity to aromatic drugs, graphene and its derivatives were proposed as fillers to mitigate burst release of chlorhexidine, ciprofloxacin, and even, natural polyphenols, such as carvacrol, from various polymer matrices [145,146,147].

### 5.2. Tissue Engineering

As previously stated, one of the most promising application fields for GO is in tissue engineering. Indeed, one of the most important paths in tissue engineering is the development of biocompatible scaffolds, able to replace highly vascularized tissues by suitably storing and delivering oxygen: this, in turn, critically affects cell proliferation and motility and, in general, the healing and functional processes of a tissue.

**Figure 10:**
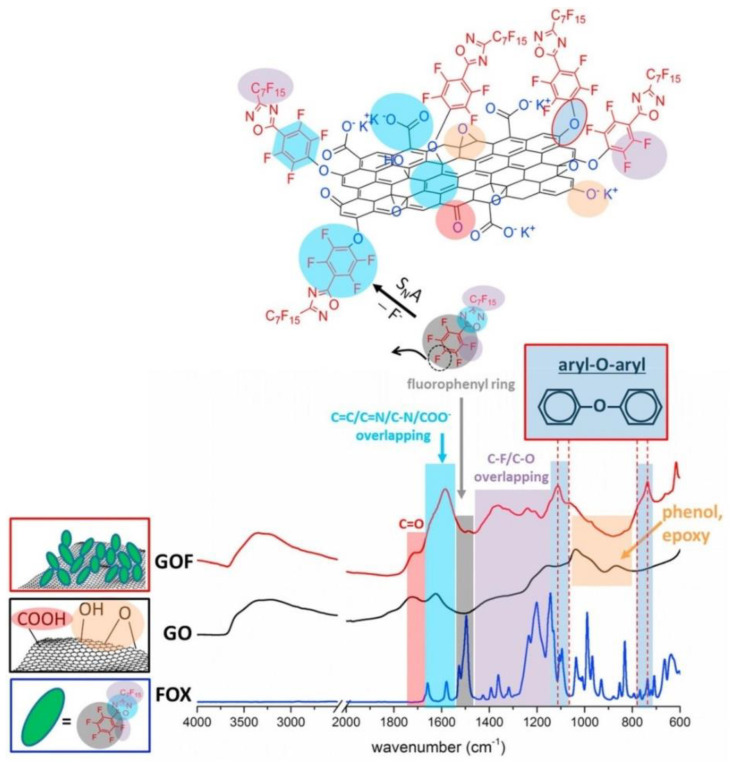
FTIR/ATR spectra of 3-pentadecafluoroheptyl,5-perfluorophenyl-1,2,4-oxadiazole (FOX), GO, and resulting nanohybrid (GOF). Reprinted with permission from [3]. Copyright Elsevier, 2017.

For instance, perfluorocarbon (PFC)-based biomaterials are well known for application in biomedical engineering [148,149,150], but, on the other hand, their hydrophobic character often makes it necessary to use cytotoxic surfactants [151]. In this context, GO has been studied as a feasible way to enhance the characteristics of PFCs. Maio et al. [3], for instance, covalently derivatized GO with the perfluorinated molecule 3-pentadecafluoroheptyl,5-perfluorophenyl-1,2,4-oxadiazole (FOX), directly onto GO or GO-silica nanohybrids (GOS) [2], by a nucleophilic aromatic substitution carried out in DMF, as pictorially schematized in Figure 10. The main advantage of this approach is the extremely high functionalization degree, considering that –OH groups are the most abundant moieties of graphene oxide. The two functionalized GOs showed good cytocompatibility, gathering hydrophilicity and biocompatibility. Furthermore, a significantly higher oxygen affinity was displayed (from 2 to 3 times higher than those of the materials currently used as oxygen reservoirs in tissue engineering).

Further biocompatible materials can be easily prepared starting from natural polymers such as chitosan and alginate [152,153,154], in order to produce multilayer films for application in wound healing, drug delivery, and tissue engineering. Some useful features of such materials include biodegradability, low toxicity, and gel-forming ability, as well as reasonably low preparation costs. For instance, Silva et al. [155] covalently functionalized graphene with *N*-benzyloxycarbonylglycine (Z-Gly-OH), in order to produce films through layer-by-layer deposition of chitosan and alginate chitosan. They found that the obtained films, beyond being cytocompatible, exhibited enhanced mechanical and electrical performance in comparison to the same systems without the functionalized graphene. These results suggest good potential for biomedical applications such as wound healing and regenerative medicine [155].

Another important and increasingly attractive goal is the development of bio-resorbable scaffolds for regenerating bone/cartilage tissues [40]. In fact, traditional therapies involving implants and bone grafts show several limitations and shortcomings, because of their invasiveness and scarce availability of raw materials. Hence, bone tissue engineering has recently emerged as a viable path for treating damaged or diseased bone cartilage tissues, and one of the preferred options to meet the requirements is starch. Starch is well known as a natural polysaccharide consisting of a large number of glucose units, linked by glycosidic bonds, and it is produced by several plants as energy storage [156].

Wu et al. [157] introduced starch into nanosized graphene oxide (nGO), thus developing a bioactive 3D porous scaffold. GO itself was achieved from starch by microwave-assisted conversion into GO nanodots; the starch and nGO were then coupled via esterification, and the porous, fully starch-based scaffolds were constructed by freeze drying. Such porous nanohybrids are promising for applications in bone/cartilage tissue engineering, since they seemed to favor the formation of large amount of hydroxyapatite in simulated body fluid and also allow controlling of the porosity level and water sorption ability by working on the content of nGO.

However, applications in biomedical engineering obviously do not include only porous scaffolds; one interesting path is represented, for instance, by nanofibers, which can be used for producing scaffolds as well.

Sarvari et al. [158], for instance, showed how to prepare electrospun nanofibers of polycaprolactone (PCL) containing reduced graphene oxide (rGO), useful for manufacturing of scaffolds capable of gathering good cytocompatibility and suitable mechanical properties.

Massoumi et al. [159] prepared covalently functionalized chlorinated GO with a graft copolymer of poly(2-hydroxyethylmethacrylate) (PHEMA) and ε-caprolactone (CL) GO-g-[P(HEMA-g-Cl)]. Electrospinning technique was then used to manufacture biocompatible and electrically conductive scaffolds for regenerative medicine.

Apart from covalent functionalization, however, also non-covalent functionalization can find some application: GO is prone to form hydrogen bonds with collagen, by virtue of its oxygenated groups, thus enabling the easy and green fabrication in the water of GO-coated hybrid materials suitable for tissue engineering applications [160].

## 6. Water Treatment

One of the most significant contributions to water pollution is related to the increasing use of dyes in many sectors, including the textile and pharmaceutical industries, thus demanding an increasing attention towards water treatment and demand for effective, functional materials to be used in such operations. In particular, some of the main pollutants for water resources are cationic dyes and metal ions, which are harmful for flora and fauna, scarcely biodegradable, and difficult to remove [161,162]. A suitable removal method could be adsorption process. However, a suitable wastewater treatment system must meet several standards, including satisfactory levels of adsorption, efficiency, and cost effectiveness [163]. Obviously, the selection of the most suitable sorbent is a critical point. Among the possible options, GO is widely considered as broad-spectrum sorbent for aqueous contaminants, relying on the coexistence of sp^2^-aromatic and sp^3^-oxygenated domains, along with defects and nanocavities [164]. Indeed, while its hydrophilicity, granted by –COOH, epoxy, and –OH groups [165], makes it compatible with water treatment operations, its sp^2^ graphenic skeletal renders GO exploitable for adsorption of dyes [166] or for formation of metal ion complexes [167].

### 6.1. Dyes Removal

Currently, polymeric hydrogels containing either graphene or GO have attracted much attention from the academia, thanks to their special properties that are particularly suitable to water treatment [168]. In fact, polymeric hydrogels are crosslinked, hydrophilic polymer chains, capable of swelling while not dissolving in water.

In this context, Soleimani et al. [169] prepared a nanocomposite hydrogel based on GO and cellulose nanowhiskers that showed outstanding absorption capabilities in removing cationic dyes such as methylene blue (MB) and Rhodamine B (RhB), with equilibrium state reached in just 15 min. In addition, the sample proved to be easily recoverable and re-usable.

Another intriguing application involves ionic liquids (ILs), which are regarded as suitable, ecologic, high-performance solvents for removal of organic dyes and heavy metals by either liquid−liquid extraction [170,171] or adsorption [172,173] but, on the other hand, pose significant problems related to the secondary pollution caused by their difficult recovery. In particular, recent literature, while reporting about ionic liquid gels as soft materials able to adsorb cationic dyes (such as RhB and MB), pointed out that their ease of recovery is not always attainable [174,175].

Zambare et al. [176] prepared an aerogel by amidation-aided coupling of methylimidazolium IL with GO and subsequent freeze-drying. Such sponge revealed high removal performance toward azo-dye direct red 80 (DR80). More in details, much higher adsorption rate and equilibrium adsorption capacity were detected, in comparison to unmodified GO and commercial samples of activated carbon. Furthermore, an almost complete removal of DR80 was achieved in aqueous solution, with the sponge even showing an excellent reusability.

### 6.2. Metal Ions Removal

It is well known that heavy metals, such as Pb, Cd, and radioactive ones, represent serious threats for human health [177,178]. Among the several techniques to decontaminate wastewater from heavy metal ions, sorption is characterized by easy practicability and cost effectiveness [179].

Some studies demonstrate the convenience of GO-based materials as sorbents for recovering metal ions from aqueous solutions.

For instance, Li et al. [180] reported a feasible strategy to prepare self-assembled Chitosan/Sulfydryl/GO composites, which were successfully tested for removing Cu(II), Cd(II), and Pb(II) both in single- and multi-metal ions systems.

Pan et al. covalently functionalized GO with primary amine derivative bearing quaternary ammonium tails [181], demonstrating that GO modification imparted strong enhancement of sorption performance, especially for removing Th(IV) and U(VI) ions.

### 6.3. Organic Molecules Removal

**Figure 11:**
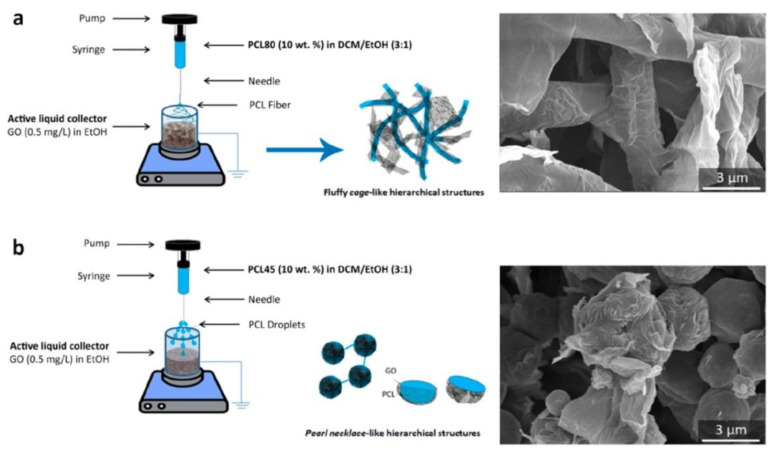
Pictorial representation of the processing-structure relationship together with SEM micrographs describing the surface texture of GO-coated PCL80 (**a**) and PCL45 (**b**). Reprinted with permission from [164]. Copyright 2020, American Chemical Society.

Owing to its peculiar structure, GO is particularly affine to organic alcohols, such as phenolic compounds. While GO free nanoparticles display a well-known ability to remove phenols from water, the latest design guidelines in terms of sorbents for water treatment indicated reusability and energy-saving separation as crucial prerequisites for minimizing environmental and economic impact. Maio et al. functionalized PCL with GO nanosheets via dry-jet-wet electrospinning onto an active liquid collector [164]. As visible in Figure 11, this novel processing method allows for the generation of peculiar polymer templates decorated by GO sheets. Non-covalent functionalization is driven by electrostatic forces, since negatively charged GO lamellae tend to wrap around positively charged PCL fibers. Such hierarchical structures proved to remove and recover phenol moieties from wastewater through low energy operations, thus paving the road to next generation sorbents, inspired by circular economy and zero-waste concepts.

### 6.4. Desalination

GO and its derivatives were lately explored in membrane technology for desalination, owing to their fouling resistance and mechanical robustness coupled with excellent performance in terms of water flux and salt rejection [182]. Fouladivanda et al. [183] integrated superhydrophobic octadecylamine functionalized rGO (ODA-rGO) sheets into PVDF-HFP membranes via electrospinning. The resulting materials displayed outstanding performance and stability, with salt rejection values of 99.99% for 4 days. In a similar study, Goel et al. [184] functionalized GO with brominated poly phenylene oxide to prepare cross-linked nanocomposite anion exchange membranes. Ultrafast water evaporation can be accomplished by exploiting functionalized GO. In this perspective, highly stable and ultrapermeable nanohybrids based on zeolitic imidazolate framework-8 (ZIF-8) nanocrystals and GO were constructed via ice templating and subsequent in situ crystallization of ZIF-8 at the GO edges [185]. For the same purpose, Chen et al. [186] fabricated subnanometer porous membranes based on GO conjugated with carbonized chitosan.

## 7. Catalysis Engineering

Heterogeneous catalysis requires solid supports for the catalysts, capable of providing cost effectiveness, ease of handling/recovery, and sustainability [187,188,189]. The morphochemical features of GO make it quite interesting for the immobilization of a number of catalysts and organo-catalysts [190], metal-based nanoparticles [191], as well as inorganic acids and bases [190]. On the basis of the available literature, the research on homogeneous catalysts anchored onto GO is still at an early stage [192]. Recent available papers concern mainly on the synthesis of new heterogeneous nanocatalysts based on the GO platform.

Porahmad et al. [193] covalently functionalized GO with 1,1,3,3-tetramethylguanidine (TMG) to construct a catalyst that showed to be highly efficient, with reaction conversion yields up to 99%, recyclability (up to seven times without significant deterioration), and applicability at high temperatures.

Bhanja et al. [194] prepared a copper-decorated GO that showed remarkable catalytic activity for C–S coupling, meant to obtain aryl thioether products and good yield stability for up to six cycles at least.

### 7.1. Synthesis for Pharmaceutical Industry

Pd is a the most used catalyst for the large-scale synthesis of most of drugs [40]. On the other hand, the homogeneous catalysis route has some severe drawbacks, related to the toxicity of the Pd-based catalysts, along with their time-consuming, expensive recovery from the homogeneous reaction systems. A possible way to get round this separation-related issues is to rely on heterogeneous catalysis, using GO substrates.

Fath et al., for instance, grafted cyclometallated palladium complexes onto the GO surface [195], obtaining supported catalysts with a higher performance than homogeneous counterparts and good recyclability.

Nancy et al. [196] anchored gold nanoparticles onto GO sheets. Noteworthy is the fast and green method that the authors adopted for preparing such nanohybrids that serve as promising catalysts for reducing 4-nitrophenol. Briefly, laser-induced ablation of an Au foil was carried out in a GO aqueous dispersion, leading to graphene oxide lamellae decorated by Au nanoparticles.

Huang et al. covalently immobilized 3-aminopropyl-triethoxysilane moieties onto GO [197], obtaining a catalyst with high catalytic performance in the Knoevenagel condensation and suitable reusability.

Acharya et al. [198] prepared phosphate functionalized GO, thus obtaining a catalyst, which proved to be particularly suitable to microwave-aided three-component Biginelli condensation reactions, including the synthesis of 3,4-dihydropyrimidin-2(1H) and 4,6-diarylpyrimidinones molecules, which were efficiently catalyzed with 96% yield and remarkable recyclability.

### 7.2. Green Chemistry

It is widely known that one of the main causes behind global warming and climate change is represented by CO_2_ [199]. On the other hand, CO_2_ may be “recovered” and transformed into valuable chemicals such as cyclic carbonates, and for the production of polar solvents, electrolyte components in lithium ion batteries, and monomers for manufacturing polycarbonates [199]. An example of a promising and potentially “green” way to recover CO_2_ is its cycloaddition with epoxides, and thus, several catalysts for this process have been developed [199], among which ionic liquids (ILs) are quite efficient [200,201]; unfortunately, their homogeneous nature is a critical disadvantage in terms of catalyst recovery.

Consequently, the literature reports some studies meant to analyze and overcome this issue. In particular, Xu et al. [202] successfully functionalized GO with imidazolium-based ionic liquids (ILs) through covalent condensation reactions, providing high catalytic activity in the solvent-free cycloaddition reactions of CO_2_ to propylene oxide, with yields up to 96%.

One of the main purposes of green chemistry is to reduce the damages caused by misconceived use of toxic agrochemicals, related to the steadily increasing world agricultural production [203]. For instance, many pesticides and insecticides based on organophosphates (OP); therefore, researchers such as Hostert et al. proposed a detoxification method that relies on GO-based nanocatalysts covalently functionalized with 1-(3-aminopropyl)imidazole groups (GOIMZ) [204]. The obtained nanocatalyst, in powder form, was tested for the destruction the toxic pesticide Paraoxon, attaining good removal yields and recoverability/recyclability. Furthermore, the feasibility of obtaining GOIMZ films was proved, thus giving a product with remarkably better handling features.

### 7.3. Biocatalysis

The plenty of oxygen-containing functionalities, along with the large surface area and dispersibility in aqueous media, make GO an ideal substrate for immobilizing enzymes [205]. This strategy has been extensively investigated to construct biosensors (as discussed above in this review paper) and nano-biocatalysts [206].

Effective fabrication routes of nano-biocatalysts may involve the enzyme immobilization via layer-by-layer deposition in multilayered systems [207]. In this regard, Patila et al. prepared a multi-layer nanobiocatalytic system, anchoring laccase from Trametes versicolor onto GO [208]. Such a nanohybrid showed enhanced thermal stability (up to 60 °C) and biocatalytic activity higher than that of the free enzyme, especially toward the oxidation of anthracene and pinacyanol chloride. Moreover, such nano-assembly displayed good reusability, with an activity retention close to 100% after five cycles.

In parallel, Wang et al. [209] prepared magnetic composites by decorating GO-chitosan (GO-CS) 3D templates with magnetite (Fe_3_O_4_) nanoparticles. More in detail, chitosan (CS) was covalently attached onto GO via amidation, performed in PBS at room temperature for 6 h. The resulting 3D network was then decorated with Fe_3_O_4_ using FeCl_3_·6H_2_O by exploiting an autoclaved solvo-thermal reaction in ethylene glycol for 8 h. The ternary GO-CS-Fe_3_O_4_ composite, relying on the unique combination of high surface area (imparted by GO), abundant presence of amino, and hydroxyl functional groups of CS and magnetic features of Fe_3_O_4_ nanoparticles, was successfully utilized to anchor *Candida rugosa* lipase (CRL) by different routes, including electrostatic adsorption, covalent binding, and metal-ion affinity interactions. Finally, the enzymatic activities of free and immobilized lipase were tested towards hydrolysis of olive oil, and the outcomes remarked not only the enhanced pH and thermal stability of immobilized CRL, but even an activity stronger than that of free enzymes. Among the three pathways followed to immobilize CRL, covalent bonding gave the best results in terms of reusability.

**Figure 12:**
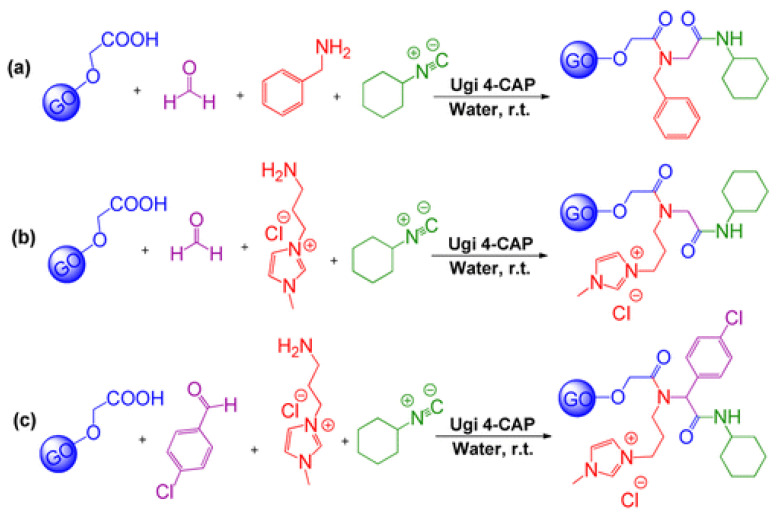
Carboxylated graphene sheets functionalized with small molecules via four-component condensation to afford (**a**) hydrophobic MFGC, (**b**) hydrophilic MFGC, and (**c**) amphiphilic MFGC. Reprinted with permission from [210]. Copyright American Chemical Society, 2016.

Rezaei et al. [210] reported the covalent functionalization of GO by Ugi four-component assembly process (Ugi 4-CAP), involving the simultaneous anchoring of amine, aldehyde, isocyanide, and acid compounds onto GO lamellae by a one-pot reaction. The resulting multifunctionalized GO composites (MFGC) were provided with tailored surface wettability, as illustrated in Figure 12. Such combinatorial synthesis enabled the development of multifunctionalized GO composites (MFGC) capable of covalently immobilizing *Bacillus thermocatenulatus* lipase (BTL), thus enhancing the biocatalytic activity of free enzyme.

Similarly, Vineha et al. covalently immobilized horseradish peroxidase (HRP) onto reduced GO (RGO) aiming to enhance the performance of the enzyme in phenol removal, in terms of kinetics, activity, stability, and reusability [211]. HRP was covalently attached onto RGO via amidation, carried out in PBS at 4 °C for 24 h, using glutaraldehyde as a cross-linker. After the immobilization, both catalytic constant and efficiency of HRP increased by 6.5 and 8.5 times, respectively, providing the enzyme with excellent reusability (70% of the initial activity retained after 10 cycles). The removal efficiency of the immobilized HRP was 100% for high phenol concentration (2500 mg/L) against the 55% of the free HRP. Notably, the immobilization endowed HRP with higher stability toward variations of pH and temperature.

Taken together, all these results testify that GO can serve as an excellent platform for the immobilization of a broad range of organocatalysts, thus enhancing their activity, chemical and thermal stability, and reusability.

## 8. Conclusions and Future Directions

The extended conjugated lattice of graphene, along with its ease of derivatization, make it extremely appealing as a building block for the next-generation materials. In this review, we examined both covalent and non-covalent derivatization strategies adopted to tune and optimize the features of graphene, in order to satisfy the most recent requests in the field of advanced applications, which practically encompass all the engineering sectors, including energy, electronic, biomedical, environmental, and catalysis engineering, and their combination, as in the case of sensors for clinical diagnostics or for the control of environmental and food quality.

The outstanding number of publications herein reviewed, along with the ensuing wealth of possible applications recently proposed, confirm the ground-breaking aspect of such a nanomaterial. Nevertheless, this intense research mainly involves small-scale and pioneering processes. Therefore, future challenges must be addressed, and the progress of nanotechnology will be reasonably crucial, in order to grant the fully commercialization of graphene and related materials. Apart from technological challenges, determination of graphene oxide structure at molecular level, as well as the in-depth understanding of how some key features of graphene materials, including electrical and thermal properties, biocompatibility, and toxicity, vary upon oxidation degree and size, are expected to be another critical factor.

## Data Availability

Not applicable.
